# Integration of ultrasound‐guided motion management into proton therapy treatment plans for ventricular tachycardia non‐invasive radio ablation

**DOI:** 10.1002/acm2.70213

**Published:** 2025-08-29

**Authors:** Eleonora Rossi, Alfredo Mirandola, Nicoletta Basla, Elisabetta Bonzano, Mario Ciocca, Andrea Cisarri, Luca Maria Colombo Gomez, Riccardo Di Liberto, Adriano Garonna, Laura Mantovani, Ester Orlandi, Andrea Pella, Antonio Sanzo, David Alberto Santos Hernandez, Adele Valentini, Luca Vicini Scajola, Roberto Rordorf, Viviana Vitolo

**Affiliations:** ^1^ Medical Physics Unit, Clinical Department CNAO Foundation Pavia Italy; ^2^ Radiology Unit‐Diagnostic Imaging I, Department of Diagnostic Medicine Istituto di Ricovero e Cura a Carattere Scientifico (IRCCS) Fondazione Policlinico San Matteo Pavia Italy; ^3^ Department of Radiation Oncology Istituto di Ricovero e Cura a Carattere Scientifico (IRCCS) Fondazione Policlinico San Matteo Pavia Italy; ^4^ Department of Medical Physics Istituto di Ricovero e Cura a Carattere Scientifico (IRCCS) Fondazione Policlinico San Matteo Pavia Italy; ^5^ Scuola di specializzazione in Fisica Medica Università degli studi di Milano Milan Italy; ^6^ EBAMed SA Geneva Switzerland; ^7^ Department of Clinical, Surgical, Diagnostic, and Pediatric Sciences University of Pavia Pavia Italy; ^8^ Radiation Oncology Unit, Clinical Department CNAO Foundation Pavia Italy; ^9^ Bioengineering Unit, Clinical Department CNAO Foundation Pavia Italy; ^10^ Arrhythmias and Electrophysiology Unit Division of Cardiology‐Fondazione IRCCS Policlinico San Matteo San Matteo Italy; ^11^ Instituto Nacional de Salud de El Salvador San Salvador El Salvador; ^12^ Department of Molecular Medicine University of Pavia Pavia Italy

**Keywords:** cardiac radiation, proton therapy, treatment planning, ultrasound guidance

## Abstract

**Background:**

Stereotactic arrhythmia radioablation (STAR) is an emerging, non‐invasive treatment for refractory ventricular arrhythmias. The technology requires target motion management.

**Purpose:**

We studied the integration of a novel ultrasound probe and holder for heart motion management into proton‐beam STAR treatment plans.

**Methods:**

Data were collected in eight of the 23 patients enrolled to‐date, in an ongoing prospective, multicenter, observational in silico study comparing treatment planning with photons versus proton radiotherapy in patients affected by Ventricular Tachycardia with indication for catheter ablation procedure. In such subgroup of patients hands‐free transthoracic echocardiography was performed in apical and parasternal positions, followed by planning Computed Tomography with ultrasound probe positions marked on the chest. A total of eleven targets were contoured and, for the proton therapy part, pencil beam scanning intensity‐modulated proton therapy plans were optimized, assuming cardio‐respiratory (dual) gated delivery.

**Results:**

In all eleven cases, it was possible to avoid beams intercepting the probe and the dosimetric constraints were fulfilled. In four cases, the position of the probe did not interfere with the beam angles defined a‐priori. In four cases, beam angles had to be modified to avoid intercepting the probe but the modified plan was equivalent to the a‐priori plan. In three cases, the modified plans included beams with longer penetration depth compared to the a‐priori plan, but all planning constraints were fulfilled.

**Conclusions:**

These results support the feasibility of using the novel ultrasound probe and holder for heart motion management. The technology would facilitate dedicated patient setup, monitoring and cardiorespiratory gating for STAR using proton beams.

## 1 INTRODUCTION

Stereotactic arrhythmia radioablation (STAR) is a novel, non‐invasive and promising treatment option for recurrent ventricular tachycardias (VT) by delivering stereotactic radiation beams of 20–25 Gy to the VT focus area^1^ STAR might overcome some of the major limitations of radiofrequency catheter ablation, which is the gold standard therapy for treating recurrent ventricular tachycardias. Radiofrequency catheter ablation requires general anesthesia or deep sedation and involves an invasive approach. In contrast, STAR is noninvasive and might be more suitable for patients at high risk of mortality and/or acute hemodynamic decompensation related to radiofrequency catheter ablation^2^ Photon radiation is most commonly used, but recently a number of studies have reported promising results with proton beam therapy, which has the potential to minimize dose to the surrounding organs at risk (OARs)^3^, ^4^, ^5^, ^6^, ^7^

A specific challenge with STAR is that of hitting an actively moving target and a number of studies have investigated the importance of target motion management (both cardiac and respiratory) in this specific treatment^8^, ^9^, ^10^ Various approaches to mitigate the effects of respiratory motion exist, for example, enlarging the target using motion encompassing margins^11^ forced shallow breathing with abdominal compression^12^ breath‐hold^13^ respiratory tracking using artificially implanted metallic fiducial markers or implanted cardioverter‐defibrillator (ICD) leads as surrogates^14^, ^15^ and respiratory gating using magnetic resonance imaging or external respiration signals.^16^, ^17^ These techniques have been used for tumor treatments with varying degrees of success, but their use with STAR has been associated with inaccuracies^18^ and with only modest improvements in margin reduction.

Ultrasound represents a non‐invasive, radiation‐free and fast (at least 25 Hz), candidate technology to address the specific challenges of real‐time image‐guidance for the heart. The ultrasound probe can be placed in two different positions (apical view and parasternal view) with a dedicated system for hands‐free imaging. The concept has been demonstrated to be feasible in real‐life VT patients^19^ and in a retrospective treatment planning study.^20^ The presence of a probe in the radiation‐beam path during the treatment can be managed by adjusting the beam direction, without any undue influence on treatment outcomes. However, prospective studies have been lacking.

We here report results from a dosimetric comparison of treatment plans with cardiorespiratory‐gated proton‐beam STAR VT with adjunctive CardioKit in eight patients enrolled in the on‐going prospective, multicenter, observational CARA‐VT (Non‐invasive CArdiac RAdioablation for Ventricular Tachycardia) study.

## 2 METHODS

CARA‐VT is an on‐going prospective, multicenter, observational study comparing the dosimetry of proton and photon STAR treatment with single (respiratory only) and dual (cardiorespiratory) gated delivery.^21^, ^22^ The study includes twenty‐three patients with an indication for transcatheter ablation; either for VT or for frequent premature ventricular contractions. The current analysis is related to the sub‐population of eight patients, which were imaged with ultrasound. The study protocol was approved by the local Ethical committee. All patients signed a study‐specific informed consent form. In the following sections, proton plan doses are given as RBE (Relative Biological Effectiveness)‐weighted doses.

### 2.1 Hands‐free ultrasound imaging

The CardioKit system (EBAMed SA, Geneva, Switzerland) is a novel, ultrasound‐based image‐guidance system for target monitoring and gated irradiation. The system provides real‐time soft tissue images and dual gating, using one single probe. The combination of cardiac (ECG) and respiratory gating could potentially reduce the extent of the target margin expansion, allowing for better OAR sparing.^17^ The CardioKit system primarily relies on image registration to calculate the extent of image displacement with reference to an image acquired in the same phase of the cardiac contraction cycle. The system is a developmental device.

**FIGURE 1:**
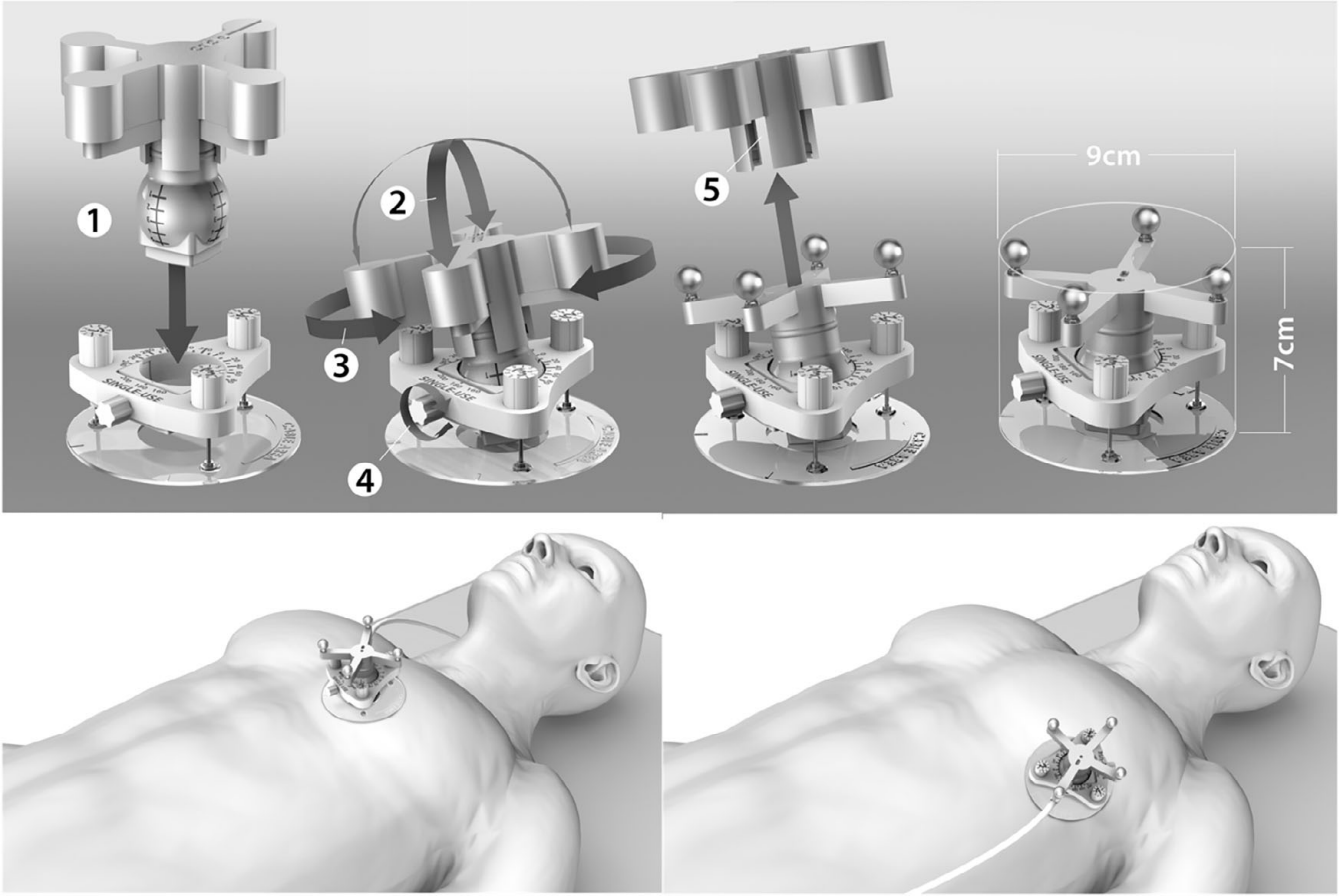
CardioKit hands‐free ultrasound imaging. Upper panel: probe positioning. The probe holder with skin‐adhesive surface and probe cover is fixed on the chest and the probe is inserted into the holder (1). The probe can be manually oriented in space with three degrees of freedom (2–3) and the skin pressure is adjusted with three pressure‐adjustment knobs. When the image is optimal, the probe is locked into position by turning the locking knob (4) and the probe cover (5) is removed. Lower panel: schematic drawing of a patient in supine position with the probe positioned in apical (right‐hand image) or parasternal (left‐hand image) positions.

Transthoracic echocardiography was performed with the patient in supine position with the probe and hands‐free imaging chest support applied in either the apical (typical diagnostic 2‐/4‐chamber apical view) or the parasternal (typical diagnostic parasternal long‐axis view) position as described in Figure 1.

### 2.2 Simulation imaging

Following ultrasound imaging for each probe position, the probe was removed but probe holders remained in place. As part of the routine diagnostic workflow for patients with ventricular arrhythmias and structural heart disease undergoing VT ablation, each patient received an ECG‐gated Computed Tomography (CT) scan. A 64 slice Canon Aquilion One Model TSX‐301A CT scanner (Canon Medical Systems Europe B.V, Netherlands) was used, in breath‐hold with 0.6 mm slice thickness, with and without contrast medium, and with the field‐of‐view centered on the heart. The diastolic cardiac phase was reconstructed at 80% of the cardiac R‐R interval (i.e., the duration between two consecutive R‐waves of the Electrocardiogram).

As a necessary condition to proceed with treatment plan optimization, every patient received an additional CT in breath‐hold of the entire thoracic area. Aiming to limit radiation exposure to patients, the basal CT resulted in a lower resolution if compared with the ECG‐gated CT (0.9 mm pixel spacing; 2 mm slice thickness).

In order to combine the anatomical information recorded under different acquisition protocols, and to obtain suitable data for the treatment planning system, the small field‐of‐view ECG‐gated acquisition of the diastolic cardiac phase was merged to the corresponding thoracic CT volume. This procedure and the following operations have been carried out in Matlab (Matlab, The MathWorks Inc. R2023b Natick, Massachusetts). Pixel spacing and slice thickness of ECG‐gated CTs were rescaled through a linear interpolation of corresponding Hounsfield Unit values, and the resulting volume was overlapped to the basal CT. In case of small but non‐negligible misalignments of bony structures, we proceeded with a manual registration (3 degrees of freedom, on the main axes [X,Y,Z]) of the vertebrae, in order to preserve the one‐to‐one voxel correspondence and to simplify the subsequent process of HU “overriding”. To minimize unrealistic artifacts coming from respiratory motion during the ECG‐gated CT, we decided to exclude the spine, ribcage and sternum from the volume of interest. Consequently, only the cardiac region area including the surrounding soft tissues was considered for the generation of a synthetic dataset, obtained by replacing the corresponding HU values (“overriding”) of the rescaled ECG‐gated over the basal CT. The selection of the appropriate region of interest was performed by manually drawing a square box that included the heart and surrounding tissues and excluded bones. For each patient dataset, a final qualitative check of the correctness of the soft tissue alignment was performed by clinical experts.

### 2.3 Electro‐anatomical mapping and contouring

**FIGURE 2:**
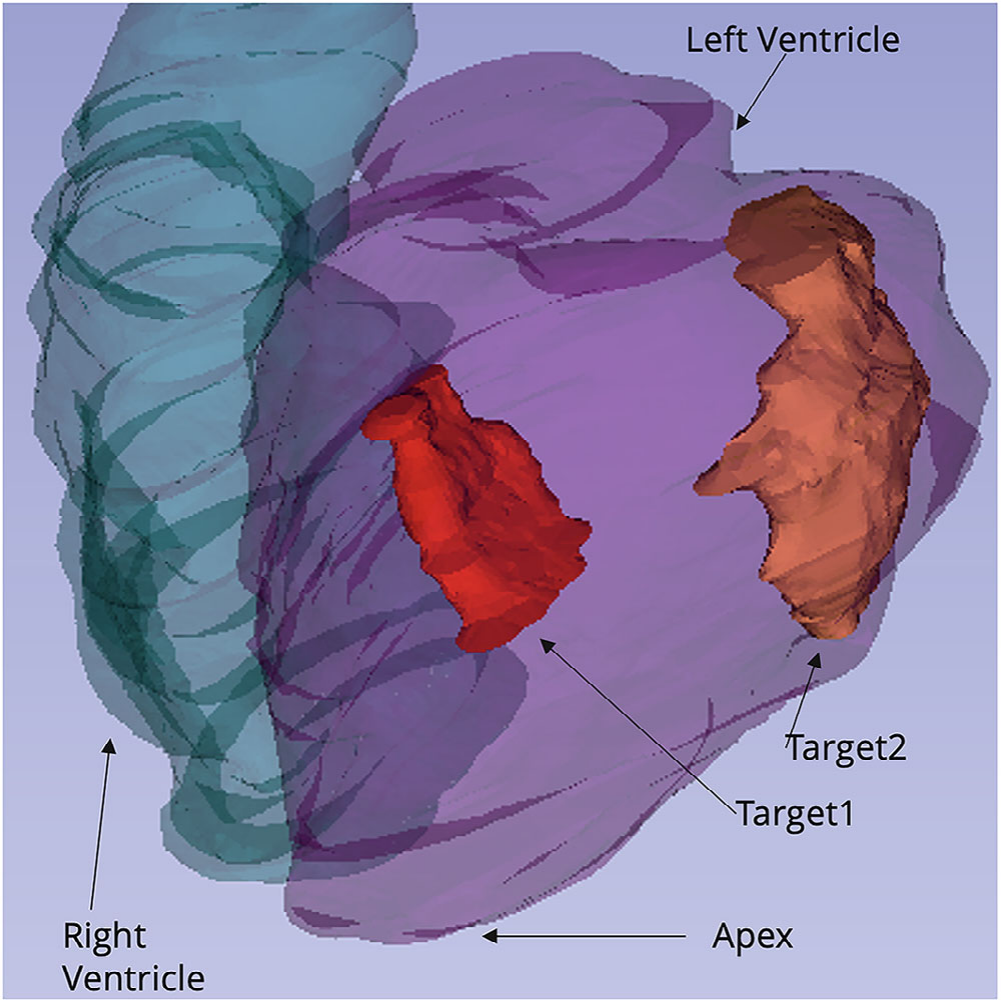
Example of a patient with two independent targets. The patient had a large scar encompassing the whole septum and the anterior wall. Two separate targets were defined because at the endocardial mapping, two separate interest areas were identified: one more septal (“target 1”) and one more antero‐lateral (“target 2”).

A 3D volumetric map of the cardiac chambers was constructed using the CARTO3 (Biosense Webster, USA) system. This information was used to contour a clinical target volume (CTV), which was subsequently mapped onto the CT images with a small field of view. The target position was determined to fit the American Heart Association (AHA) 17‐segment model, following the current clinical practice for STAR.^22^ In two patients, multiple independent targets were identified due to the presence of a large ventricular scar and more than one area of interest identified on the electroanatomical map. Figure 2 illustrates the situation for one of the two patients.

These were treated as independent targets, making up to a total of 11 targets for eight patients. No cardiac or respiratory motion was included during target contouring, assuming that delivery was performed with the CardioKit cardiorespiratory (dual) gating system.

Contouring of targets and heart substructures was performed on the small FOV diastolic CT with contrast medium following recommendations from the literature.^23^, ^24^, ^25^, ^26^ A rigid registration was then performed between the contrast enhanced CT and the full FOV synthetic CT in order to map the contours and allow treatment planning. Besides the extracardiac organs (lungs, esophagus, aorta, pulmonary arteries, stomach, bowel, liver), this study focuses also on non‐targeted cardiac substructures, such as the specialized myocardium (valves, coronary arteries and pericardium).

### 2.4 Treatment planning

Treatment plans were optimized with the Raystation v. 11B (Raysearch Laboratories, Sweden) treatment planning system (TPS) and the clinical proton beam model currently in use at CNAO, simulating a full rotational gantry when selecting the beam geometry. The TPS dose grid had a resolution of 2 mm, with a Monte Carlo v. 5.5 dose‐calculation engine.

Dose prescription to the target was 25 Gy(RBE) in a single fraction, as supported by the literature.^27^, ^28^, ^29^ A constant RBE of 1.1 was considered, following the current clinical practice.^30^ No overdoses above 107% of the prescribed dose were allowed. The target coverage constraints were: D_95%_ ≥23.75 Gy and D_1%_ < 27.5 Gy(RBE). Constraints for OARs followed those used for STAR in the recent RAVENTA study^31^ and reported in Table 1, with the addition of D_max _< 12.4 Gy as a dose recommendation for the stomach.^32^, ^33^

**TABLE 1:** Treatment planning constraints for OAR.

Aorta	Dose limitation: D_max_ ≤ 20.0 Gy Minor protocol deviation: 20 Gy < D_max_ ≤ 25 Gy Major protocol violation: D_max_ > 25 Gy
Left coronary arteries	Dose limitation: D_max_ ≤ 14.0 Gy Minor protocol deviation: 14 Gy < D_max_ ≤ 20 Gy Major protocol violation: D_max_ > 20 Gy
Superior vena cava	Dose recommendation: D_50%_ ≤ 0.6 Gy
Left atrium	Dose recommendation: D_max_ ≤ 4.4 Gy
Whole heart minus ITV/PTV	Dose recommendation: D_50%_ ≤ 5 Gy
Esophagus	Dose limitations: D_max_ ≤ 14.5 Gy and V_9 Gy_ ≤ 1 cc Minor protocol deviations: D_max_ ≤ 19 Gy, D_1 cc_ ≤ 14.5 Gy and V_9 Gy_ ≤ 4 cc Major protocol violations: D_max_ > 19 Gy; D_1cc_ > 14.5 Gy; V_9 Gy_ > 4 cc
Stomach‐Duodenum	Dose limitations: D_max_ ≤ 12.4 Gy and D10 cc < 9 Gy
Trachea	Dose limitations: D_max_ ≤ 15 Gy and V_10 Gy_ ≤ 1 cc Minor protocol deviations: D_max_ ≤ 20 Gy, D_1 cc_ ≤ 15 Gy and V_10 Gy_ ≤ 4 cc Major protocol violations: D_max_ > 20 Gy; D_1 cc_ > 15 Gy; V_9 Gy _> 4 cc
Bronchial tree	Dose limitations: D_max_ ≤ 15 Gy and V_10Gy_ ≤ 1 cc Minor protocol deviations: D_max_ ≤ 20 Gy, D_1 cc_ ≤ 15 Gy and V_10 Gy_ ≤ 4 cc Major protocol violations: D_max_ > 20 Gy; D_1 cc_ > 15 Gy; V_9 Gy_ > 4 cc
Spinal canal	Dose limitations: D_max_ ≤ 7 Gy and V_6 Gy_ ≤ 0.1 cc Minor protocol deviations: D_max_ ≤ 8 Gy, V_6Gy_ ≤ 1 cc Major protocol violations: D_max _> 8 Gy; V_6Gy_ > 1 cc
Skin	Dose limitations: D_max_ ≤ 14.4 Gy and V_10 Gy_ ≤ 10 cc Minor protocol deviations: D_max_ ≤ 16 Gy, V_14.4 Gy_ ≤ 10 cc Major protocol violations: D_max_ > 16 Gy; V_14.4 Gy_ > 10 cc
Whole lungs	Dose limitations: V_100%_ − V_7Gy_ ≥ 1500 cc (V_7 Gy_ remaining volume > 1500 cc) and D_5%_ ≤ 20 Gy and D_50%_ ≤ 3.5 Gy Minor protocol deviations: V_100%_ − V_7Gy_ ≥ 1000 cc (V_7 Gy_ remaining volume > 1000 cc), D_6.5%_ ≤ 20 Gy and D_50%_ ≤ 5 Gy Major protocol violations: V_100%_ − V_7 Gy_ < 1000 cc (V_7 Gy_ remaining volume < 1000 cc), D_6.5%_ > 20 Gy and D_50%_ > 5 Gy
ICD	Dose limitations: D_max_ ≤ 0.5 Gy and blocked from primary beam irradiation Minor protocol deviation: 0.5 Gy < D_max_ ≤ 1.0 Gy Major protocol violation: D_max_ > 1.0 Gy

Plans used pencil‐beam scanning intensity modulated proton therapy with at least two entrance beams at different angles depending on target position and patient anatomy. All the plans were optimized with the following robustness parameters: 3 mm isotropic margin for setup uncertainties and 3% uncertainties in proton range (−3%, 0%, 3%). The robust plans were evaluated over 21 scenarios, resulting from the combination of the uncertainties mentioned above. Each plan was accepted if all the scenarios satisfied the minimum criteria of V_95%_ > 90%.

### 2.5 Treatment plan evaluation

To compare the quality of the plans with and without the probe, the target coverage (D_95%_) was evaluated and the following quantities for OARs were used: the residual volume receiving > 7 Gy (V_100%_‐V_7Gy_) for the lung and the maximal dose (D_0.1cc_) for all the other OARs. The dose constraint values were used to compare the plans with relative percentage values.

Firstly, we optimized a “reference plan” choosing the optimal beam geometry independently from the probe position. One alternative plan was then optimized for each probe position (apical or parasternal location) if one or both the probes were intercepted by the beam entrances of the reference plan. Each treatment plan was internally reviewed and qualitatively evaluated with a score ranging between 0 and 3, assigned based on consensus between two medical physicists. A score of 3 represented the best‐case scenario: the probe position did not intercept the beams’ path of the reference plan, and no adaptation was needed specifically for the probes. The score 2 was assigned if an alternative plan was needed, with small deviations from the reference plan geometry and resulting in unaffected dosimetric constraints and dose distribution. A score of 1 was assigned to plans where the beam angles had to be adapted, all dosimetric constraints were still satisfied but the beam geometry and low‐dose irradiation of healthy tissues were not optimal compared to the reference plan. A plan where it was not possible to avoid beams intercepting the probe and fulfilling the dosimetric constraints was assigned a score of 0.

In this feasibility study there was no a‐priori hypothesis and data were analyzed descriptively. Results are presented as numbers and percentages, means ± standard deviation or as medians and interquartile ranges, as appropriate.

## 3 RESULTS

### 3.1 Patients

A total of eight patients (seven men) were enrolled between July 2023 and April 2024. Table 2 provides more details about patients’ characteristics.

**TABLE 2:** Patients’ characteristics.

Median age (years)	67 (range 43–82)
Median body‐mass index (kg/m^2^)	25 (range 20–33)
Patients with ventricular tachycardia (#)	5
Patients with premature ventricular contractions (#)	3
Patients with structural heart disease (#)	7 (4 ischemic cardiomyopathy + 3 cardiomyopathy with preserved coronary arteries)
Mean left ventricular ejection fraction	40%

Only one patient did not present structural cardiac abnormalities and all patients had a clinical indication to transcatheter ablation for ventricular arrhythmias.

### 3.2 Target sizes and locations

Target sizes ranged from 0.2 to 15.8 cc (Table 3). The spatial positions of targets within the left ventricle covered all segments of the AHA model, except segment 1 (basal anterior) and 14 (apical septal). Ten out of 17 segments in the model were covered twice (Table 3). One target was in the aortic valve region and could not be represented using the AHA model. No adverse events occurred during ultrasound and CT imaging.

**TABLE 3:** Target sizes and locations within the left ventricle. The location of the target is shown according to the 17‐segment AHA model, when applicable. PVC and VT stand for premature ventricular contractions and ventricular tachycardia respectively.

Patient‐target identifiers→	1‐a	1‐b	1‐c	2‐a	3‐a	4‐a	4‐b	5‐a	6‐a	7‐a	8‐a
Clinical target volume (cc)	8.0	14.2	3.3	5.6	10.6	3.6	17.3	0.2	15.8	3.5	7.9
Location	2‐3‐8‐9	10‐13‐15‐16‐17	7–12	2	7‐12‐13	3	5–6	Aortic valve	4‐10‐11	15–16	5–6
Clinical arrhythmia	PVC	PVC	PVC	PVC	VT	VT	VT	PVC	VT	VT	VT

### 3.3 Scoring of plan quality and impact of probe position

**FIGURE 3:**
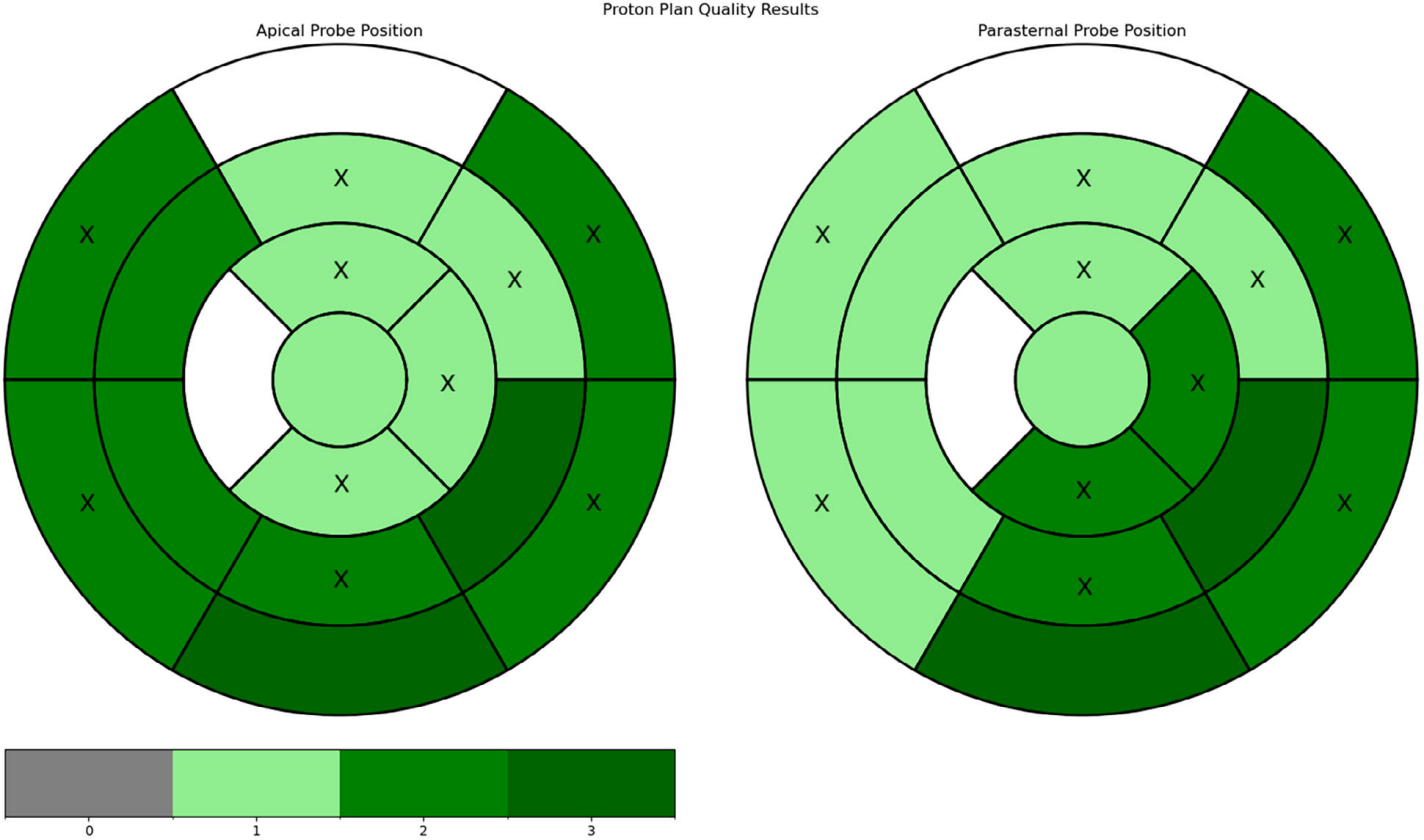
Spatial distribution of treatment‐plan quality scores in the left ventricle according to the AHA 17‐segment model. The quality scores of treatment plans for each segment, from 4 (highest) to 0 (lowest) are indicated by colors. White fields represent the two segments within the left ventricle which could not be covered in the model. For the segments where two plans were available (marked with “X” on the figure), the average score is used. Left‐hand image: apical probe position; right‐hand image: parasternal probe position.

It was feasible to generate treatment plans that fully met all target and OAR constraints for every case and probe position. (Figure 3).

The data appeared consistent across target segments with only the apical inferior‐lateral and mid infero/anterolateral regions showing more than 1 point difference in quality scores between neighboring segments. The basal inferior and inferolateral regions had the highest score for both probe positions. Lowest plan quality was found in the apical and mid‐anterior regions for the apical probe position and in the basal and mid septal regions for the parasternal probe position.

In four cases, the position of the probe did not interfere with the a‐priori defined beam angles. In four cases, the beam angles had to be modified to avoid intercepting the probe but the plan had the same score as the one without the probe. In the remaining three cases, the modified plans had a lower quality score compared with the plan without the probe but fulfilled all planning dose constraints. No zero score was assigned and the probe position did not relevantly affect the scores. In one case there was a two‐point difference and in four cases a one‐point difference. In the three cases with equal score for both positions, the apical position was found to be ideal to maximize the distance between beam entrance and probe or to avoid beams passing closer to the lung.

As a control, the modeling and treatment‐plan analyses were re‐run utilizing single‐gating (respiratory only) delivery, where an Internal Target Volume for cardiac motion was delineated based on the diastolic phase reconstruction of the 4D‐CT (described in the Methods) and an additional 30%R‐R (systole) reconstruction of the 4D‐CT (processed in the same way to obtain a systolic synthetic CT). The results were essentially the same as for the dual‐gated plans.

### 3.4 Dosimetric results

**FIGURE 4:**
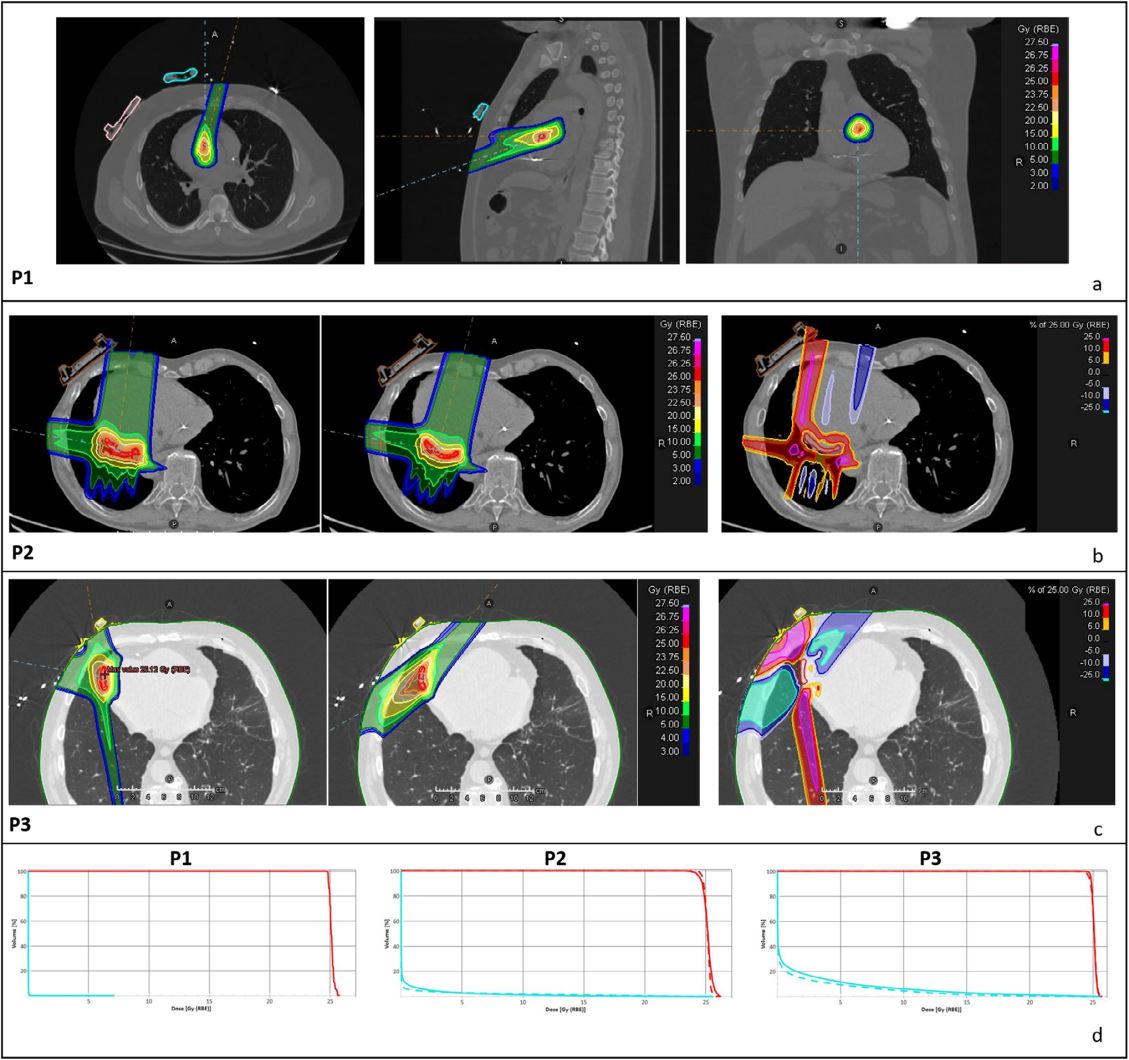
Representative treatment plans. (a) Best‐case scenario: example of a treatment plan that was not affected by the probe placement. The apical and parasternal probes are delineated by a pink and blue contour respectively and the beams’ geometry is represented by the orange and blue dashed lines for the transversal, sagittal and coronal view. (b) Mid‐range‐case scenario: example of a treatment plan with a slightly modified beam geometry to avoid intercepting the apical probe (brown contour). From left to right: dose distribution of the reference plan, dose distribution of the edited plan and dose difference between the two. The difference is expressed in percentage relative to the dose prescription. A negative difference means that the dose is higher compared to the reference plan. (c) Worst‐case scenario: this treatment plan demonstrates major modifications in beam geometry. To avoid the probe, a larger volume of the left lung receives medium‐high doses. Furthermore, a greater air gap exists between the beam entrance and the target compared to the reference plan, potentially reducing the treatment plan's robustness. (d) Dose volume histograms (DVHs) corresponding to the treatment plans shown above. The solid line represents the nominal plan, and the dashed line shows the modified plan. Red and blue colors denote the target and lungs statistics, respectively. Each DVH is labeled with a patient ID (P1, P2, P3) to match it with its corresponding dose distribution.

Three representative dose distributions from different patients in the study population and the corresponding DVHs are shown in Figure 4. A quantitative comparison of dosimetric data for plans with and without modifications due to the probe location is provided in Table 4 for both target and critical structures.

**TABLE 4:** Treatment plans comparison. Dose differences between plans adapted to consider the probe position and plans not considering the probe are reported as percentages of a reference value. A positive value indicates a higher dose value for the plan considering the probe. The reference value represents the major protocol violation value. If unavailable, twice the dose recommendation/limitation is reported. The statistics only include the adapted plans (thus the wording “subset”).

Structure of interest	Dosimetric parameter	Subset first interquartile	Subset median	Subset third interquartile
Target	ΔD_95%_/25 Gy (%)	−0.6	−0.2	0.9
Ascending Aorta	ΔD_0.1cc_/25 Gy (%)	0.0	0.0	0.1
Descending Aorta	ΔD_0.1cc_/25 Gy (%)	0.0	0.0	0.8
Aortic Arch	ΔD_0.1cc_/25 Gy (%)	0.0	0.0	0.0
Left Anterior Descending Artery	ΔD_0.1cc_/20 Gy (%)	−0.2	2.8	17.2
Left Main Coronary Artery	ΔD_0.1cc_/20 Gy (%)	0.0	0.0	0.0
Circumflex Coronary	ΔD_0.1cc_/20 Gy (%)	−0.1	0.0	0.0
Left Atrium	ΔD_0.1cc_/8.8 Gy (%)	−1.5	0.1	1.3
Right Atrium	ΔD_0.1cc_/8.8 Gy (%)	−0.7	−0.1	2.0
Superior Vena Cava	ΔD_0.1cc_/1.2 Gy (%)	−0.2	0.0	0.0
Inferior Vena Cava	ΔD_0.1cc_/1.2 Gy (%)	−0.1	0.0	0.0
Aortic valve	ΔD_0.1cc_/20 Gy (%)	0.0	0.0	0.7
Mitral valve	ΔD_0.1cc_/20 Gy(%)	−2.0	0.1	1.6
Tricuspid valve	ΔD_0.1cc_/20 Gy(%)	0.0	0.0	1.3
Pulmonary valve	ΔD_0.1cc_/20 Gy(%)	0.0	0.0	0.0
Esophagus	ΔD_0.1cc_ /19 Gy (%)	−5.6	−0.2	0.0
Stomach	ΔD_0.1cc_/24.8 Gy (%)	0.0	0.0	0.1
Trachea	ΔD_0.1cc_/20 Gy (%)	0.0	0.0	0.0
Bronchial tree	ΔD_0.1cc_/20 Gy (%)	0.0	0.0	0.1
Spinal canal	ΔD_0.1cc_/8 Gy (%)	0.0	0.0	0.0
Skin	ΔD_0.1cc_/16 Gy (%)	−0.2	0.0	0.9
Whole lungs	Δ (V_100%_‐V_7Gy_)/1000 cc (%)	−6.8	−0.6	0.7
ICD	ΔD_0.1cc_/1 Gy (%)	0.0	0.0	0.0
Whole heart minus ITV/PTV	ΔD_0.1cc_/10 Gy (%)	−0.8	−0.2	0.3

The interquartile values for each structure across all adapted plans were reported in Table 4. The first interquartile ranged from −6.8% (“whole lungs”) to 0.0%, the median ranged from −0.6% to +2.8% (“Left Anterior Descending Artery”) and the third interquartile ranged from 0.0% to +17.2% (“Left Anterior Descending Artery”). The Supplementary Materials section provides additional details (S1).

## 4 DISCUSSION

To our knowledge, this is the first prospective study of the impact of an ultrasound‐based gating probe on VT treatment. The results support the feasibility of integrating the CardioKit heart motion gating device into treatment plans for proton‐beam STAR ablation of VT. Apical as well as parasternal probe positions were possible in all cases, with slightly better plan quality for the apical probe position. When comparing doses to cardiac and extra‐cardiac structures, no overall relevant dosimetric differences appeared between plans with and without the probe, except for the skin. Thus, the analysis supports the expectation that use of ultrasound imaging during proton‐beam STAR ablation of VT will allow for internal target volume margin reductions.^20^

### 4.1 Cardiac motion management

STAR represents an emerging advancement in the treatment of recurrent VT, particularly for patients who do not respond to conventional therapies. However, the fairly high ablative dose of around 25 Gy is usually prescribed, and as a consequence there is a need to ensure focused delivery of radiation and minimized exposure to OARs. Proton beam STAR is an emerging alternative to photon beam therapy, with the potential advantage of more precise radiation dose delivery. However, it is crucial to be cautious in order to minimize the risks associated with uncertainties in the range of the proton beam's penetration into tissue. To address this issue, we adopted robust planning strategies: from choosing a favorable beam geometry (avoiding non‐homogeneous entrances when feasible) to using robust planning objectives on target coverage and OARs preservation. In this setting, image guidance can play a role as well, and is particularly relevant for cardiac applications where cardiac‐ and respiration‐induced target motion or setup misalignments could potentially have an impact on the effectiveness of the procedure and the risk to OARs, due to the high dose delivered in a single fraction.^9^, ^17^, ^34^

There is no standard for motion management so far. A number of ultrasound‐based motion tracking systems are at different stages of development, most of which have been employed in oncology applications. The challenge was to overcome shortcomings such as the anatomical deformations^34^ the inconsistency between users with freehand systems^35^, ^36^ and the lack of real‐time monitoring during beam delivery.^37^ These limitations have been addressed in CardioKit by using automatic interpretation of the images with a proprietary deep‐learning algorithm and dedicated hardware for reproducible placement of the ultrasound probe.^19^

### 4.2 Dose constraints

The parameters selected for the evaluation correspond to what was used in studies treating VT patients with STAR^1^ although this is a new line of therapy and there is a general lack of consensus on treatment planning parameters, such as target coverage and OARs dose limits for single fraction delivery.^38^ Currently, heart dose constraints during thoracic radiation therapy are based on the dose received by the whole cardiac volume. To date, there is limited data on the correlation between the dose received by cardiac substructures (valves, coronary arteries, etc.) and potential side effects. A correlation between mean dose and decline in left ventricular (LV) ejection fraction and LV end‐diastolic volume 3 months after proton‐beam treatment has been reported in swine models^39^ but no specific dose constraints have been validated for cardiac substructures.

### 4.3 Strengths and weaknesses

The treatment plans covered a large variety of target positions in the left ventricle, excepting only two of the 17 segments in the AHA model.^40^ Probe positioning was based on real‐time ultrasound imaging.

Being a feasibility study, there are some weaknesses, most notably the relatively modest number of patients and targets. The findings and conclusions apply only to the specific probe and hands‐free imaging holder used in the procedures and cannot be extrapolated to other ultrasound‐based systems. In addition, we generated a synthetic CT image combining the diastolic‐phase gated small field‐of‐view CT with the breath‐hold large field of view CT required for treatment planning. We are aware that this step may have introduced small residuals and uncertainties in Hounsfield unit values (despite the fact that the correctness of soft tissue anatomy registration and alignment have been qualitatively verified by a clinical expert for each patient dataset), still it was our intention to minimize the additional imaging dose to the patients enrolled in the study.

Another peculiar aspect in the generation of the synthetic CT is related to the manual delineation of the regions of interest. This step was necessary to avoid unrealistic gradients in the tissue density along the beam path, especially near the ribcage and sternum. Clinical experts made a qualitative assessment of image quality during treatment plan optimization, but no critical observations concerning the scope of this study arose.

Although real treatments require a large field‐of‐view cardiac‐gated CT, we believe that the limitations concerning the synthetic CTs do not significantly impact our findings, given the scope of the study.

## 5 CONCLUSIONS

This prospective analysis supports the feasibility of integrating CardioKit ultrasound‐based gating for heart motion management into treatment plans for STAR using proton beams. Provided regulatory approval, the system promises to be a useful and uncomplicated tool to enhance the performance and safety of STAR in clinical practice.

## AUTHOR CONTRIBUTIONS

Eleonora Rossi: conception and design, acquisition of data, analysis and interpretation of data. Alfredo Mirandola: conception and design, acquisition of data, analysis and interpretation of data. Nicoletta Basla: conception and design, acquisition of data. Elisabetta Bonzano: conception and design, acquisition of data. Mario Ciocca: conception and design. Andrea Cisarri: acquisition of data. Luca Maria Colombo Gomez: conception and design, acquisition of data, analysis and interpretation of data. Riccardo Di Liberto: conception and design. Adriano Garonna: conception and design, acquisition of data, analysis and interpretation of data. Laura Mantovani: conception and design, acquisition of data, analysis and interpretation of data. Ester Orlandi: conception and design. Andrea Pella: conception and design, acquisition of data. Antonio Sanzo: acquisition of data. David Alberto Santos Hernandez: acquisition of data. Adele Valentini: conception and design, acquisition of data. Luca Vicini Scajola: acquisition of data. Roberto Rordorf: conception and design, analysis and interpretation of data. Viviana Vitolo: conception and design, acquisition of data, analysis and interpretation of data

## CONFLICT OF INTEREST STATEMENT

Adriano Garonna is an employee of EBAMed SA. The other authors report no conflict of interest.

## ETHICS STATEMENT

The study protocol was approved by the local Ethical committee at the San Matteo Foundation.

## Supporting information

Supporting Information
