# Supplementary material for: Integration of ultrasound‐guided motion management into proton therapy treatment plans for ventricular tachycardia non‐invasive radio ablation
Source: J Appl Clin Med Phys. 2025 Aug 29;26(9):e70213. doi: 10.1002/acm2.70213 (PMC12396888; doi:10.1002/acm2.70213)
Supplement: Supplementary file 1 — Supporting Information [file ACM2-26-e70213-s001.docx]

## Supplementary Materials

|  | Case Identifier→ | 1-a | | 1-b | | 1-c | | 2-a | | 3-a | | 4-a | | 4-b | | 5-a | | 6-a | | 7-a | | 8-a | |
| --- | --- | --- | --- | --- | --- | --- | --- | --- | --- | --- | --- | --- | --- | --- | --- | --- | --- | --- | --- | --- | --- | --- | --- |
|  | Plan→ | A | P | A | P | A | P | A | P | A | P | A | P | A | P | A | P | A | P | A | P | A | P |
| Structure of interest ↓ | Dosimetric parameter ↓ |  |  |  |  |  |  |  |  |  |  |  |  |  |  |  |  |  |  |  |  |  |  |
| Target | ΔD_95%_ / 25 Gy [%] | 0.2 | 0.0 | 5.5 | 9.2 | -2.4 | -2.3 | -0.5 | -1.0 | -0.6 | -0.4 | 0.0 | 1.8 | 0.0 | 0.0 | 0.0 | 0.0 | 0.0 | 0.0 | 1.1 | 0.0 | -0.5 | 0.0 |
| Ascending Aorta | ΔD_0.1cc_ / 25 Gy [%] | -0.5 | 0.7 | 1.1 | 9.1 | -0.2 | -0.2 | 0.0 | 0.1 | 0.0 | 0.0 | 0.0 | 0.0 | 0.0 | 0.0 | 0.0 | 0.0 | 0.0 | 0.0 | 0.0 | 0.0 | 0.0 | 0.0 |
| Descending Aorta | ΔD_0.1cc_ / 25 Gy [%] | 4.0 | 6.7 | 1.1 | 9.1 | 0.0 | 0.0 | 0.0 | 0.0 | 0.0 | 0.0 | 0.0 | 0.0 | 0.0 | 0.0 | 0.0 | 0.0 | 0.0 | 0.0 | 0.0 | 0.0 | -17.0 | -5.5 |
| Aortic Arch | ΔD_0.1cc_ / 25 Gy [%] | 0.0 | 0.0 | 0.0 | 0.0 | 0.0 | 0.0 | 0.0 | 0.0 | 0.0 | 0.1 | 0.0 | 0.0 | 0.0 | 0.0 | 0.0 | 0.0 | 0.0 | 0.0 | 0.0 | 0.0 | 0.0 | 0.0 |
| Left Anterior Descending Artery | ΔD_0.1cc_ / 20 Gy [%] | 12.6 | 20.3 | -0.2 | 3.2 | -1.0 | -10.7 | -0.1 | 0.0 | -3.8 | 21.6 | 0.0 | 26.5 | 0.0 | 0.0 | 0.0 | 0.0 | 0.0 | 0.0 | 2.3 | 0.0 | 10.9 | 18.7 |
| Left Main Coronary Artery | ΔD_0.1cc_ / 20 Gy [%] | 0.0 | 0.1 | 0.2 | 0.0 | 0.0 | 0.0 | 0.0 | 0.0 | 0.0 | 0.0 | 0.0 | 0.0 | 0.0 | 0.0 | 0.0 | 0.0 | 0.0 | 0.0 | 0.0 | 0.0 | 0.0 | 0.0 |
| Circumflex Coronary | ΔD_0.1cc_ / 20 Gy [%] | -0.1 | 0.0 | 7.1 | 0.1 | -0.1 | 3.6 | 0.0 | 0.0 | 0.0 | 0.0 | 0.0 | 0.0 | 0.0 | 0.0 | 0.0 | 0.0 | 0.0 | 0.0 | 0.0 | 0.0 | -12.4 | -1.4 |
| Left Atrium | ΔD_0.1cc_ / 8.8 Gy [%] | -1.8 | 1.4 | 13.4 | 18.3 | -2.4 | -2.3 | 0.8 | -0.6 | 0.1 | 0.1 | 0.0 | 0.0 | 0.0 | 0.0 | 0.0 | 0.0 | 0.0 | 0.0 | 0.0 | 0.0 | -15.8 | 1.8 |
| Right Atrium | ΔD_0.1cc_ / 8.8 Gy [%] | -0.8 | 23.4 | 31.3 | -12.5 | -0.1 | -0.1 | -11.5 | 6.6 | 0.0 | -0.1 | 0.0 | -5.1 | 0.0 | 0.0 | 0.0 | 0.0 | 0.0 | 0.0 | 0.0 | 0.0 | 2.6 | -0.4 |
| Superior Vena Cava | ΔD_0.1cc_ / 1.2 Gy [%] | 0.0 | -0.2 | 0.2 | -0.9 | -0.2 | -0.2 | 0.0 | 0.0 | 0.0 | 0.0 | 0.0 | 0.0 | 0.0 | 0.0 | 0.0 | 0.0 | 0.0 | 0.0 | 0.0 | 0.0 | 0.1 | 0.0 |
| Inferior Vena Cava | ΔD_0.1cc_ / 1.2 Gy [%] | -1.2 | -4.6 | 35.6 | 19.3 | -0.1 | -0.1 | 0.0 | 0.0 | 0.1 | -0.1 | 0.0 | 0.0 | 0.0 | 0.0 | 0.0 | 0.0 | 0.0 | 0.0 | 0.0 | 0.0 | 0.0 | -0.3 |
| Aortic valve | ΔD_0.1cc_ / 20 Gy [%] | 1.4 | 7.5 | 0.9 | 1.1 | -0.1 | -0.1 | 0.0 | 0.0 | 0.0 | 0.0 | 0.0 | 0.1 | 0.0 | 0.0 | 0.0 | 0.0 | 0.0 | 0.0 | 0.0 | 0.0 | 0.1 | 0.0 |
| Mitral valve | ΔD_0.1cc_ / 20 Gy[%] | -3.0 | 2.0 | 14.3 | 3.6 | -2.6 | -4.4 | -0.1 | 0.1 | 0.0 | 0.1 | 0.0 | 0.2 | 0.0 | 0.0 | 0.0 | 0.0 | 0.0 | 0.0 | 0.0 | 0.0 | -7.2 | 4.1 |
| Tricuspid Valve | ΔD_0.1cc_ / 20 Gy[%] | 1.3 | 4.9 | 15.3 | -3.2 | 0.0 | 0.0 | 17.9 | -9.7 | 0.0 | 0.0 | 0.0 | 1.2 | 0.0 | 0.0 | 0.0 | 0.0 | 0.0 | 0.0 | 0.0 | 0.0 | 0.5 | -0.1 |
| Pulmonary Valve | ΔD_0.1cc_ / 20 Gy[%] | 0.0 | 0.1 | 0.1 | 0.0 | 0.1 | 0.0 | 0.0 | -0.4 | 0.0 | 0.0 | 0.0 | 0.0 | 0.0 | 0.0 | 0.0 | 0.0 | 0.0 | 0.0 | 0.0 | 0.0 | 0.0 | 0.0 |
| Esophagus | ΔD_0.1cc_ /19 Gy [%] | -6.1 | -16.1 | 13.6 | 12.7 | 0.0 | 0.0 | -10.8 | -0.4 | 0.0 | 0.0 | 0.0 | -7.3 | 0.0 | 0.0 | 0.0 | 0.0 | 0.0 | 0.0 | 0.0 | 0.0 | -4.2 | -3.8 |
| Stomach | ΔD_0.1cc_ / 24.8 Gy [%] | -0.3 | 0.9 | 0.4 | -0.7 | 0.0 | 0.1 | 0.0 | 0.0 | 0.0 | 0.0 | 0.0 | 0.5 | 0.0 | 0.0 | 0.0 | 0.0 | 0.0 | 0.0 | 0.0 | 0.0 | 0.0 | 0.0 |
| Trachea | ΔD_0.1cc_ / 20 Gy [%] | 0.0 | 0.0 | 0.0 | 0.0 | 0.0 | 0.0 | 0.0 | 0.0 | 0.0 | 0.0 | 0.0 | 0.0 | 0.0 | 0.0 | 0.0 | 0.0 | 0.0 | 0.0 | 0.0 | 0.0 | 0.0 | 0.0 |
| Bronchial Tree | ΔD_0.1cc_ / 20 Gy [%] | 0.0 | 0.7 | 0.1 | 0.1 | -0.8 | 0.0 | 0.0 | 0.0 | 0.0 | 0.2 | 0.0 | 0.0 | 0.0 | 0.0 | 0.0 | 0.0 | 0.0 | 0.0 | 0.0 | 0.0 | -0.2 | 0.0 |
| Spinal Canal | ΔD_0.1cc_ / 8 Gy [%] | 0.0 | 4.4 | 0.8 | 0.4 | 0.0 | 0.0 | 0.0 | 0.0 | 0.0 | 0.0 | 0.0 | 0.0 | 0.0 | 0.0 | 0.0 | 0.0 | 0.0 | 0.0 | 0.0 | 0.0 | -0.1 | -0.3 |
| Skin | ΔD_0.1cc_ / 16 Gy [%] | -3.4 | -1.8 | 2.2 | 1.2 | 5.7 | 5.0 | 0.0 | 0.0 | 0.0 | -0.4 | 0.0 | 0.0 | 0.0 | 0.0 | 0.0 | 0.0 | 0.0 | 0.0 | 0.0 | 0.0 | 0.0 | -0.2 |
| Whole lungs | Δ (V_100%_-V_7Gy_)/1000 cc [%] | -0.2 | -0.5 | -9.5 | -8.9 | 1.3 | 1.4 | -0.6 | 0.1 | -8.5 | -9.1 | 0.0 | -1.3 | 0.0 | 0.0 | 0.0 | 0.0 | 0.0 | 0.0 | -1.8 | 0.0 | 3.5 | 0.9 |
| ICD | ΔD_0.1cc_ / 1 Gy [%] | 0.0 | 0.0 | 0.0 | 0.0 | 0.0 | 0.0 | 0.0 | 0.0 | 0.0 | 0.0 | 0.0 | 0.0 | 0.0 | 0.0 | 0.0 | 0.0 | 0.0 | 0.0 | 0.0 | 0.0 | 0.0 | 0.0 |
| Whole heart minus ITV/PTV | ΔD_0.1cc_ / 10 Gy [%] | -1.6 | -0.9 | -0.5 | 0.2 | 2.7 | 2.2 | -0.8 | -0.8 | -0.2 | 0.3 | 0.0 | 1.5 | 0.0 | 0.0 | 0.0 | 0.0 | 0.0 | 0.0 | -0.2 | 0.0 | -0.8 | -0.2 |

**Table A: Treatment plans comparison.** Dose differences between plans adapted to consider the probe position and plans not considering the probe are reported as percentages of a reference value. A positive value indicates a higher dose value for the plan which is considering the probe. A: values for plans adapted for apical probe position; P: values for plans adapted for parasternal probe position. The reference value represents the major protocol violation value. If unavailable, twice the dose recommendation/limitation is reported. Cells higher than 10% or lower than -10% are highlighted in gray.
